# Feasibility of an MI-CBT ketogenic adherence program for older adults with mild cognitive impairment

**DOI:** 10.1186/s40814-022-00970-z

**Published:** 2022-01-22

**Authors:** Julia L. Sheffler, Bahram Arjmandi, Jamie Quinn, Greg Hajcak, Cynthia Vied, Neda Akhavan, Sylvie Naar

**Affiliations:** Florida State University, Tallahassee, USA

**Keywords:** Mild cognitive impairment, Intervention, Motivational interviewing, Cognitive Behavioral therapy, Adherence

## Abstract

**Background:**

The National Institutes of Health Obesity-Related Behavioral Intervention Trials model for intervention development was used to establish the feasibility and proof of concept of a motivational ketogenic nutrition adherence program for older adults with mild cognitive impairment.

**Methods:**

This was a single-arm, single-center feasibility trial. A comprehensive assessment protocol, including a clinical interview, neuropsychological testing, and genetic sequencing was used as an initial screening. Nine participants (aged 64–75) with possible amnestic mild cognitive impairment were consented for the intervention. Participants completed pre- and post-intervention neuropsychological assessments using the updated Repeatable Battery for Assessment of Neuropsychological Status. Participants tracked their macronutrient consumption using food diaries and ketone levels using urinalysis test strips daily. Mood and other psychosocial variables were collected through surveys, and qualitative exit interviews were completed.

**Results:**

100% of participants who began the trial completed the 6-week ketogenic nutrition adherence program, including completion of the pre- and post-assessments. Eight participants achieved measurable levels of ketones during the program. The average self-rated adherence across the program was 8.7 out of 10. A Wilcoxon Signed-Rank test demonstrated significant improvement in cognitive performance from baseline (median = 88) to follow up (median = 96, *Z* = − 2.26, *p* = .024). The average difference in cognitive performance from baseline to follow-up was − 7.33 (95% CI − 12.85, − 1.82).

**Conclusions:**

Results supported the feasibility for moving to the next phase and demonstrated proof of concept for the intervention. The next step is a randomized pilot trial to test clinical signals of effect compared to a control condition.

**Trial registration:**

This trial was retrospectively registered with clinicaltrials.gov on July 13, 2021. The trial number is NCT04968041.

## Key messages regarding feasibility

- While ketogenic diets have shown significant promise in reducing risk for and ameliorating the symptoms of neurocognitive disorders, adherence to this type of diet remains a significant hurdle for broader dissemination and community uptake.
- Our study demonstrates that a structured, brief program incorporating motivational interviewing and cognitive behavioral strategies may promote increased uptake and adherence for older adults at risk for neurocognitive disorders.
- Our study establishes the feasibility of this intervention design and provided important information about modifications to enhance recruitment and the intervention materials to inform future trials.

Health behavior and lifestyle interventions (e.g., nutrition, exercise, sleep) have shown significant promise in delaying onset and decreasing risk for Alzheimer’s disease [1–3]; however, there are significant challenges to implementing lifestyle preventions for Alzheimer’s disease in real-world settings [4, 5]. Some of these challenges include long-term adherence difficulties, inconsistent and poor-quality information, and lack of adaptation of materials for older adults. Ketogenic nutrition is gaining recognition in the literature as a particularly promising intervention for targeting Alzheimer’s disease and other related metabolic risks. Recent studies indicate that ketogenic nutrition targets multiple neurobiological mechanisms associated with the development of Alzheimer’s disease, such as defects in mitochondrial function, cerebrospinal fluid biomarkers, insulin signaling, and dysfunctional glucose and lipid metabolism [3, 6, 7]. Further, recent feasibility trials of ketogenic nutrition demonstrate substantial improvements in cognitive scores and mood, suggesting that ketogenic nutrition is a promising therapeutic for targeting multiple disease markers associated with Alzheimer’s disease [8–11]. Despite its promise, ketogenic nutrition is an especially strict and challenging type of nutrition for adherence, which suggests that an early translational science approach may be needed to develop an intervention that addresses the challenges related to ketogenic nutrition and other lifestyle intervention adherence among older adults.

Among existing nutrition and lifestyle approaches, interventions often exclude important motivational and long-term adherence components vital for real-world implementation. These issues may be addressed using components of existing psychological treatments. For example, motivational interviewing (MI) is a client-centered approach to therapy that uses a collaborative style to help patients resolve ambivalence and make lasting changes [12]. Additionally, cognitive behavioral therapy (CBT) has some of the strongest evidence for behavior change in its favor [13]. Yet, it is also true that many individuals do not respond to treatment, do not adhere to treatment tasks, discontinue treatment prematurely, or, after initial success, are unable to maintain change [14, 15]. Experts in both CBT and MI have suggested this may be due at least in part to CBT approaches not specifying the skills necessary to support the practitioner’s relationship with the client and do not help practitioners strengthen motivation for change at both the onset and during the course of CBT [16, 17]. Thus, integrating MI and CBT strategies may improve both initial response rates and maintenance of change after treatment is completed [18]. MI can make CBT work better.

While an integrated MI-CBT approach has gained traction in younger and middle-aged populations [19, 20], few have adapted this integrated approach for addressing behavior change in older adulthood. It is important to note, however, that MI and CBT have individually shown efficacy in older individuals. For example, a brief MI intervention was shown to increase physical activity in long-term cancer survivors compared to a non-MI control group [21], and CBT may reduce fear of falling [22], improve sleep quality, and decrease pain in older adults [23]. Given these promising findings, MI-CBT strategies can be adapted for older adults and may enhance motivation and long-term adherence to nutritional programs, such as the use of ketogenic nutrition to target cognitive and metabolic risks for AD.

### Rigorous translation to develop more potent behavioral interventions

The National Institutes of Health (NIH) Obesity-Related Behavioral Intervention Trials (ORBIT) model for behavioral intervention development [3–5] provides a useful framework for designing and preliminary intervention testing (T1 translation) prior to full-scale clinical trials to increase rigor and replicability. The model consists of two phases. Phase 1 (Define) tasks include identifying the basic behavioral and social science studies that form the foundation of what is translated into potential intervention components, specifying clinically significant milestones, and identifying intervention components from the literature that could be adapted for the new intervention. The intervention should balance efficiency with potential efficacy and be refined for specific subpopulations. Phase 2 includes preliminary testing in proof-of-concept studies (Phase 2a), feasibility pilots (Phase 2b), and small efficacy trials (Phase 2c).

Using this model, we sought to build on prior clinical efficacy trials demonstrating the effectiveness of ketogenic nutrition on improving cognitive functioning [24, 25]. First, while prior trials have shown good adherence to ketogenic nutrition during the trial, these methods are often too costly or ineffective for long-term dissemination to the community. For example, Neth and colleagues [24] provide an excellent trial demonstrating the potential efficacy of ketogenic nutrition; however, the use of individualized diet plans created by a registered dietitian is not feasible for many community members. Further, another study showed that most participants did not intend to continue this type of diet after completing the trial [25]. Thus, a low-cost and motivational program designed to enhance long-term adherence to ketogenic nutrition may help this promising intervention reach the people who may benefit most.

The goal of the current study was to design for dissemination and test the feasibility of a protocol using MI-CBT strategies to promote adherence to ketogenic nutrition in high-risk older adults. To determine feasibility of the protocol to move to pilot testing, we examined [1] recruitment uptake, [2] treatment adherence, and [3] participant retention in the trial. First, we examined the feasibility of the screening and recruitment protocol, which was designed to recruit five older adults with possible mild cognitive impairment (MCI) and an apolipoprotein E (APOE) €4 allele, and five with only MCI and no genetic risk. We expected that 20% of screened participants would be eligible based on MCI status, and 15% eligible based on APOE and MCI status combined. Next, we examined whether the program would produce good adherence to ketogenic nutrition, as evidenced by at least 80% of participants demonstrating measurable levels of urine ketones by week 6 of the program. Third, we examined the feasibility of the program to maintain high participant retention, as evidenced by a 90% completion rate. Further, we assessed acceptability of the program based on participant self-report. Finally, we assessed whether there was clinically significant improvement in our target of cognitive performance. We expected preliminary evidence that adherence to ketogenic nutrition would improve cognitive performance on the Repeatable Battery for the Assessment of Neuropsychological Status—Update (RBANS-Update). Based on the NIH ORBIT model, these hypotheses provide evidence of readiness to proceed to the next stage of clinical trial development.

## Methods

### Participant recruitment and screening

This study was a single-arm, feasibility trial completed at a single site. Participants were recruited and screened for the intervention through multiple phases of data collection. First, participants aged 55 and older were recruited from the Florida State University’s Institute for Successful Longevity participant registry to complete an initial online survey that assessed for a range of health and psychosocial information (*N* = 517). A subsample of participants who completed the survey and volunteered to participate were recruited. After signing the consent form, participants (*N* = 79) completed two, in-person appointments, which included neuropsychological assessments and a clinical interview, the Emotion Battery of the NIH Toolbox, and collection of a saliva sample for genetic sequencing for the apolipoprotein E (APOE) €4 alleles [26]. All in-person assessments and interviews were reviewed by two raters, and possible diagnoses (e.g., MCI, dementia) were assigned through consensus between a psychology resident and a licensed clinical psychologist using the Diagnostic and Statistical Manuel of Mental Disorders, 5th edition. Final inclusion criteria included individuals who met criteria for “possible” MCI, which was defined as the following: evidence of either subjective decline in memory and greater self-reported use of compensatory strategies **OR** poorer performance than expected in one or more cognitive domain **OR** limited evidence of impairment in both subjective and objective findings. The feasibility of the initial inclusion criteria and required modifications to the screening plan are discussed in the Results section. Briefly, feasibility of the recruitment strategies was determined based on the following criteria: [1] 20% of screened participants are eligible based on MCI and health status, and [2] 15% of screened participants are eligible based on combined APOE and MCI status. These target rates were based on population rates of MCI and APOE €4.

**Fig. 1:**
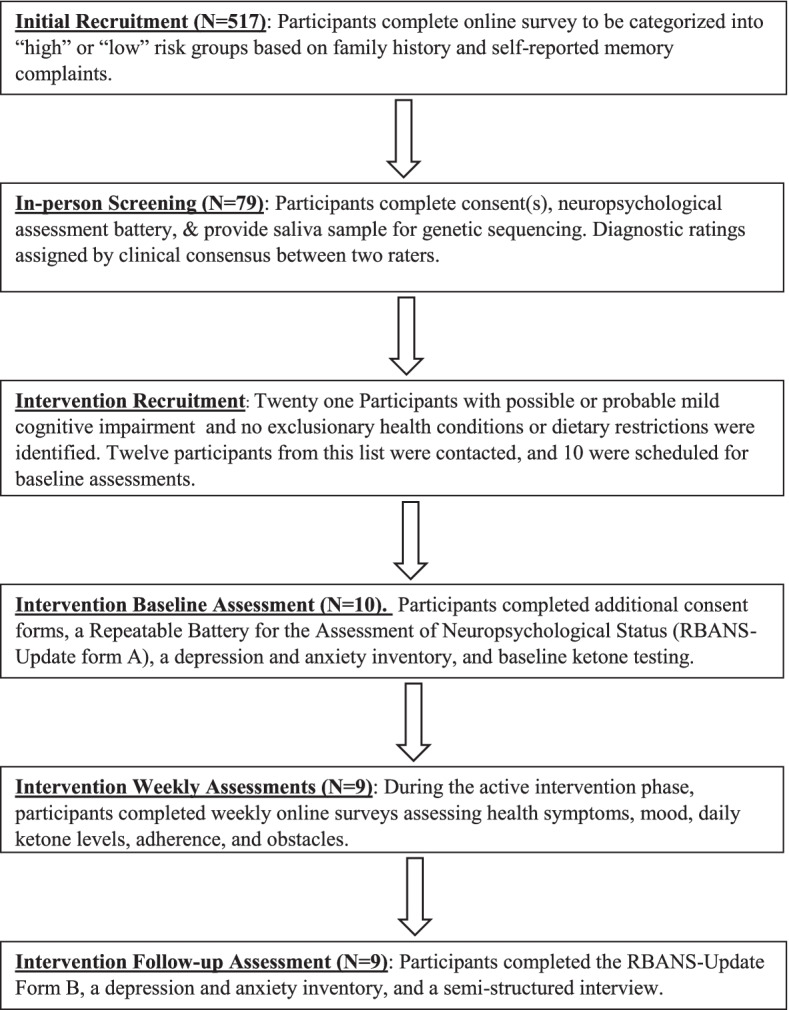
Participant recruitment and assessment flow diagram

Nineteen participants from this sample met criteria for possible MCI and did not have exclusionary health conditions. Exclusionary health conditions were defined as chronic, severe unstable health conditions that may impact an individual’s ability to significantly alter their diet, as determined by a nurse practitioner. Of the 79 screened participants, 18 were APOE €4+ (see Table 1); however, only three of these 18 individuals also met criteria for possible MCI. Participants were contacted from this list of 19 until the study *N* of 10 was reached. A target *N* of 10 was determined based on previously reported effect sizes of ketogenic interventions on cognitive outcomes [8]. Twelve individuals were contacted to assess interest in completing the MI-CBT KNA program, and 10 agreed to join the program, with the remaining two indicating that they were interested, but unable to attend the scheduled dates. Following the changes to the protocol due to COVID-19 (see below), nine of the 10 consented participants agreed to remain in the study and begin the virtual intervention. Two of the nine participants were APOE €4+ (see Fig. 1 for the participant flow diagram).

**Table 1:** Apolipoprotein E participant frequencies

Common name	rs429358	rs7412	Frequency	Percent of participants
APOE-ε1/ε3 or APOE-ε2/ε4	(C;T)	(C;T)	2	2.70%
APOE-ε2/ε3	(T;T)	(C;T)	11	14.86%
APOE-ε3/ε3	(T;T)	(C;C)	43	58.11%
APOE-ε3/ε4	(C;T)	(C;C)	16	21.62%
APOE-ε4/ε4	(C;C)	(C;C)	2	2.70%

### Saliva collection and genotyping

For APOE genotyping, saliva was collected from participants during the in-person neuropsychological assessment, and DNA was isolated using DNA/RNA Shield Saliva Collection Kits and Quick-DNA miniprep plus kit (Zymo Research Corporation) according to the manufacturer’s suggestions. DNA libraries for sequencing were generated using AmpliSeq^TM^ custom DNA panel and AmpliSeq^TM^ Library Plus for Illumina. Multiplexed libraries were sequenced on an Illumina MiSeq paired-end 250 base Nano run. DNA Amplicon (version 2.1.1; Illumina BaseSpace application) was used for variant calling using the BWA aligner to the custom DNA amplicon manifest.

### Overall intervention design and modifications due to COVID-19

#### ORBIT Phase Ia and Ib (Define & Refine)

Given prior research demonstrating the potential for ketogenic nutrition to enhance cognitive function [8, 27], we aimed to develop a structured ketogenic nutrition program for older adults with MCI to improve adherence, which in turn may further enhance cognitive function. Prior work demonstrated that MI and CBT may be especially useful for targeting adherence. Flynn [28] discusses several ways that MI can be combined with CBT. First, MI may be delivered as a brief pre-treatment to build motivation for multi-session intervention. Second, MI can be used at specific moments during CBT when discord or ambivalence arises. Third, MI can serve as an integrative framework in which other interventions, such as CBT strategies, could be delivered. Our team developed such an integrated framework for multiple behaviors [18], including nutrition [29]. Selected MI-CBT worksheets were adapted for older adults (e.g., larger font, relevant examples) with examples specific to our ketogenic nutrition intervention.

Ketogenic nutrition information was based on prior pilot studies using ketogenic and Mediterranean Ketogenic diets for older adults [8, 24, 30] and consultation with the registered dietitian and licensed nutritionist on the team. Briefly, the program involved weekly titration toward a diet consisting of 70% healthy fats, 20% proteins, and 10% carbohydrates. A titrated approach was used to reduce the risk of gastrointestinal side-effects. Participants were provided with tracking materials, optional recipes, and individual guidance during weekly meetings for making this change using nutritious foods. Participants were encouraged to individualize their meals based on their preferences; however, an emphasis was placed on foods consistent with a Mediterranean ketogenic diet (e.g., fish, olive oil, and nuts).

Once specific treatment components were delineated, the team worked to refine the treatment protocol to specifically target ketogenic nutrition adherence and related cognitive and functional outcomes. A participant workbook was developed that included the MI-CBT worksheets, weekly goals, recipes, handouts for counting and tracking macronutrients, and informational handouts about healthy ketogenic nutrition. Handouts were divided by week and reviewed as part of the weekly sessions. Weekly presentations were developed through collaboration between the team psychologist, licensed nurse practitioner, and registered dietitian. These presentations were developed to be interactive and provide participants with opportunities to interact with the group leaders and other participants for problem-solving and support, while learning how to transition to ketogenic nutrition and use CBT strategies. Given prior research demonstrating clinical improvements in adherence related to the included MI-CBT components [29] and studies demonstrating clinical improvements in cognitive functioning related to ketogenic nutrition in older adults, our team moved directly to feasibility testing in the proof-of-concept phase after developing the MI-CBT ketogenic nutrition adherence (KNA) intervention protocol (Phase IIa).

#### ORBIT Phase IIa & b (Feasibility & Proof-of-Concept)

The primary goal of the proof-of-concept phase is to use pre-post comparisons to determine if the treatment package can produce clinically significant improvements. Thus, in addition to evaluating the feasibility of the protocol in our target population, a secondary goal was to assess whether the KNA program influenced cognitive outcomes. Of note, recruitment for the trial occurred in the weeks leading up to the COVID-19 pandemic, which substantially altered the original intervention and assessment design due to restrictions on in-person human subject research. While the original protocol included in-person assessments and group meetings, collection of multiple biological samples (i.e., lipid panels, basic vitals, inflammatory biomarkers), and weekly health assessments, these in-person components were modified or removed for completion during the COVID-19 pandemic. Pre-assessment and screening appointments described below were completed in-person prior to the COVID-19 pandemic. Thus, we completed pre-intervention neuropsychological assessments with participants at baseline prior to beginning the trial; however, immediately following baseline assessments, we fully revised the protocol to include only online, video, and phone contact. We moved the KNA program to an online platform (HIPAA-compliant Zoom), and assessments were changed to online surveys and video assessments. Individual testing of the video platform was completed prior to the trial, which successfully reduced technical problems.

The KNA program was assessed as a single-arm, within-subjects clinical study with a target enrollment of 10 participants with possible or probable amnestic MCI. The protocol required participants to complete a pre-intervention assessment within 2 weeks of beginning the intervention, attend seven, one-hour intervention group sessions across 6 weeks, complete a post-assessment and interview in the final week of the intervention, and complete weekly surveys throughout the intervention. Participants were mailed materials, including the participant workbook and food, macronutrient, and ketone logs. Participants were also sent two bottles of ketone urine test strips for daily testing and materials to complete follow-up assessments. Throughout the program, participants were instructed to titrate into full ketosis across the first 4 weeks of the program by gradually reducing total carbohydrate intake and increasing healthy fat intake (e.g., fish, nuts, avocado, olive oils, etc.). Thus, participants were not expected to attain measurable ketone levels until the final weeks of the program.

This study was approved by the Florida State University Internal Review Board, and written informed consent was provided from all participants.

### Measures

#### Intervention feasibility

*Adherence* was assessed using participants’ reported morning levels of urine ketones. Participants were instructed on the use of at-home urine ketone test strips prior to beginning the trial and were asked to record their daily levels collected prior to eating breakfast each morning throughout the 6-week trial. Given that participants were expected to titrate into a state of ketosis, adherence based on ketone level was only examined in the final week of the program. Additionally, participants self-reported their weekly adherence using a 0 (not at all) to 10 (Very consistent) scale. Feasibility of the program for enhancing adherence was defined by a minimum of 80% of participants reaching measurable levels of ketosis by the final week of the program. *Retention* in the trial was assessed based on the number of sessions participants attended and completion of post-assessments. Feasibility of the program for enhancing retention was defined by retaining at least 90% of participants from baseline to follow-up.

#### Intervention acceptability

Acceptability of the intervention was determined based on qualitative feedback collected during semi-structured exit interviews in the final week of the program. These interviews were completed via HIPAA compliant Zoom with the primary investigator. The semi-structured interview included questions about aspects of the program that were most and least useful, whether participants would recommend the program, and whether participants would continue implementing the diet and skills learned during the program. An online survey was also used to collect information about acceptability, such as benefits and obstacles encountered, reported through open-ended questions, and whether the program helped them achieve their goals (0 = None, 3 = All).

#### Cognitive functioning

The Repeatable Battery for the Assessment of Neuropsychological Status – Update (RBANS-Update) Form A was administered at baseline, prior to beginning the trial [31]. To reduce potential test-retest effects, Forms B of the RBANS-Update was administered in the final week of the program. The RBANS assesses cognitive domains of immediate memory, visuospatial/constructional, language, attention, and delayed memory, and includes a total scale score. Given the small sample size and early phase of the trial, we examined total scale score mean change on the measure for clinically significant change. We also completed a paired samples *t*-test to examine pre- and post-intervention statistical signals of effect using IBM SPSS Statistics software [32].

## Results

### Sample characteristics

Nine participants were enrolled in the intervention, aged 64–75. There were two women and seven men. Four participants identified as Caucasian, two as Black, and three as Latino/Hispanic. Education levels ranged from a high school diploma to doctoral degree, and three individuals reported living in a rural area. Thus, although the sample was small, there was good representation across multiple underrepresented groups. All nine participants were included in all intervention analyses.

### Feasibility of recruitment protocol

The planned inclusion criteria for the study required modification in order to identify sufficient participants for the intervention. The first modification included expanding the criteria for MCI symptoms from a “probable” diagnosis to a “possible” diagnosis. Second, we were unable to identify sufficient overlap in participants meeting diagnostic criteria who also had a copy of the APOE €4 allele (goal *N* of 11 or 15% of screened participants). Only two participants who were APOE €4+ were included in the trial. In sum, the recruitment protocol was not feasible for successfully identifying APOE €4+ individuals with MCI. Thus, modification to the inclusion criteria in future trials to include individuals who are “at risk” or in a pre-MCI stage may increase the feasibility of these recruitment strategies. Specifically, approximately 24% of screened participants were eligible based on this expanded criterion. Thus, for a future randomized pilot study to be powered at 0.80 and *p* < .05 significance, in order to detect a large effect size (*f* = 0.4) using one-way ANOVA with fixed effects a sample size of 52 will be required. A total of 208 participants will need to be recruited for evaluation assuming approximately one-fourth of participants would be eligible for participation.

### Intervention adherence and retention

**Fig. 2:**
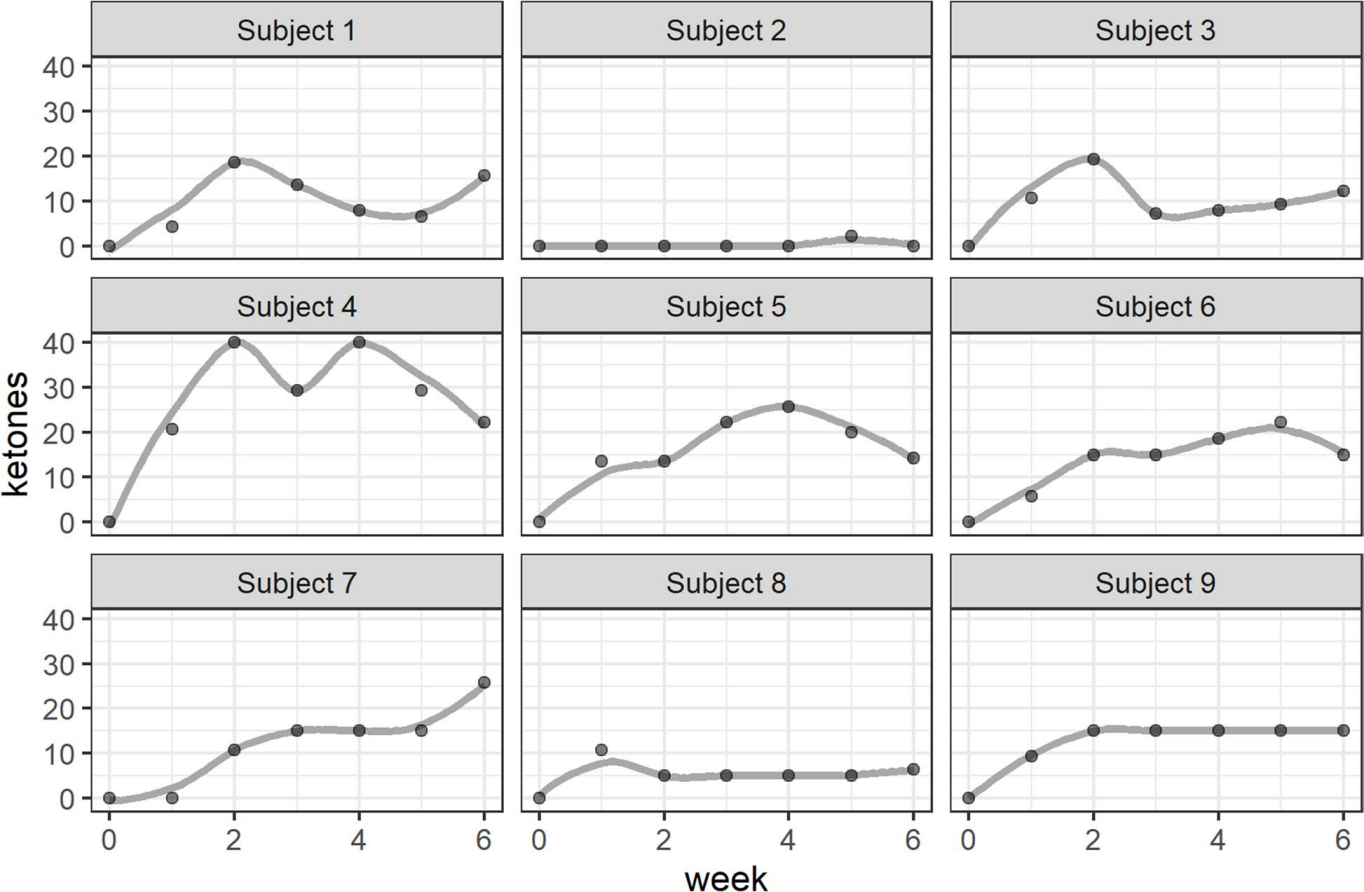
Participant weekly average urine ketone levels

Weekly average ketone levels split by subject are presented in Fig. 2. By week 6, eight of the nine participants (88.8%) reported at least one day or more with greater than trace amounts of ketone levels. The individual who was unable to obtain measurable levels of ketones self-reported high adherence. It is notable that metabolic differences may have played a role in ketone levels, as we identified a significant correlation between daily ketone level and weight (*r* = .846, *p* = .004), where higher weight was associated with more days with measurable ketone levels. Self-reported adherence to ketogenic nutrition was high (*M* = 8.56, SD = 1.87). Five participants reported “very consistent” adherence, and the lowest rated adherence was “half the time.”

In regard to retention, 100% of participants who began the intervention completed the program and post-assessments. Two participants missed one out of seven sessions due to technology challenges, such as poor internet connection and difficulty accessing the current link, that were resolved between sessions. Overall, the intervention protocol demonstrated good feasibility based on high adherence and retention rates.

### Clinical signal of effect on cognition

**Fig. 3:**
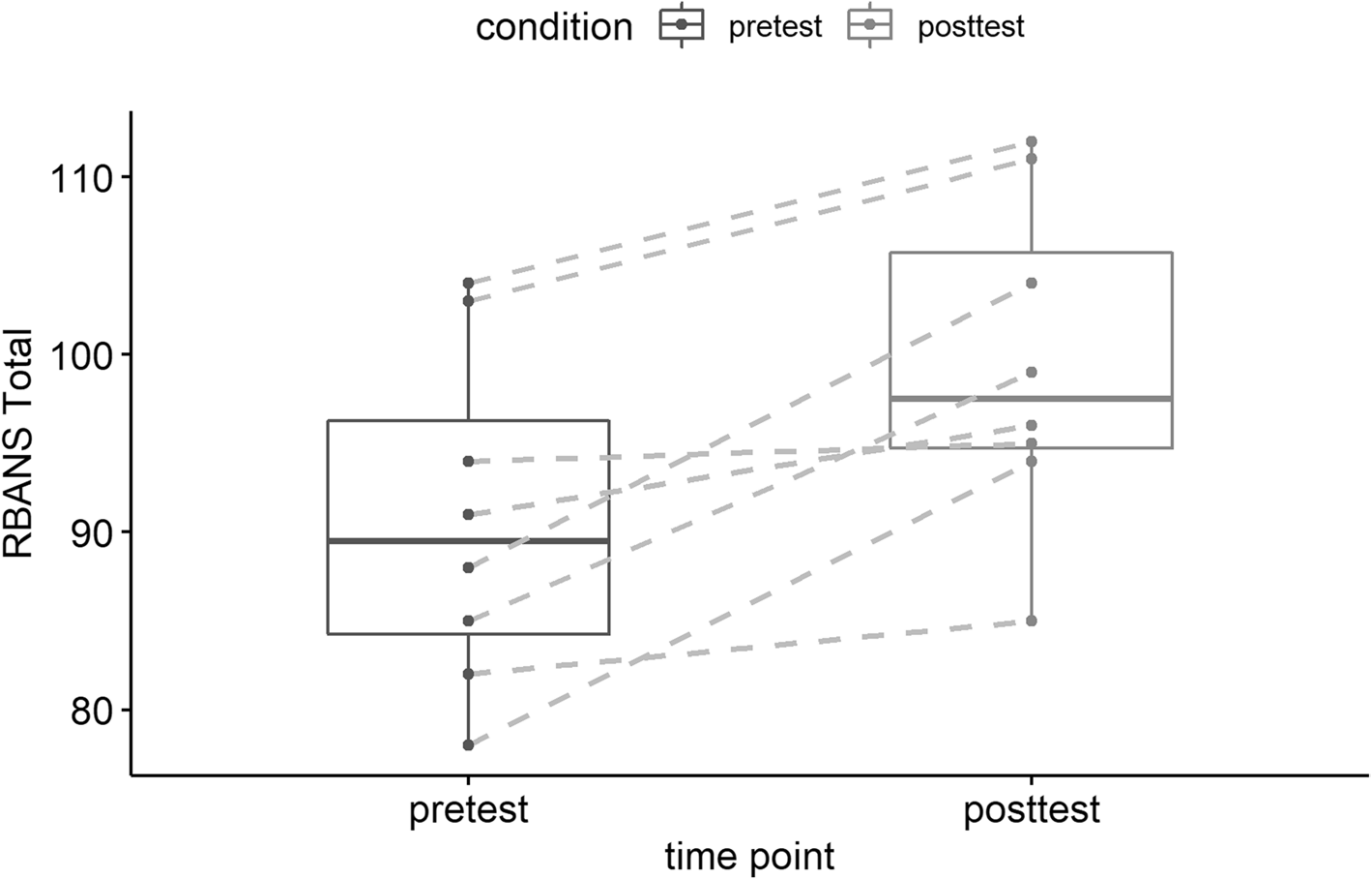
Change in the Repeatable Battery for the Assessment of Neuropsychological Status (RBANS) total score from pre-test to post-test

At baseline, participants scored in the low average range on the RBANS-Update Form A total scaled score (median = 88, range 78–104), which is consistent with MCI based on prior research [33]. After 6 weeks in the program, participants scored in the average range on the RBANS-Update Form B total scaled score (median = 96, range 85–112), demonstrating clinical improvement. A Wilcoxon Signed Ranks test indicated that this change was statistically significant, *Z* = − 2.26, *p* = .024. The average difference in cognitive performance was − 7.33 (95% CI − 12.85, − 1.82). A paired box plot of RBANS total scores is presented in Fig. 3. Of note, one participant demonstrated decline from baseline to follow-up; however, it was noted that this participant’s follow-up score should be “interpreted with caution” due to multiple interruptions during the testing session. Removal of this participant does not significantly alter the reported findings. There were no significant associations between baseline RBANS total scores and measures of adherence.

### Qualitative acceptability of the program

Qualitative feedback about the program was collected through an online survey and during semi-structured interviews. From the open-ended survey questions, participants reported benefits, such as improved sleep quality, higher energy levels, healthy weight loss, and generally “feeling better.” Participants also reported some obstacles in the survey, including craving carbohydrate-rich foods, difficulty finding “good tasting” foods, and feeling tempted by television ads for unhealthy foods. In regard to side-effects of the program, one participant reported constipation and a second participant reported unintentional weight loss. Participants were asked how well they felt the program helped them achieve their health-related goals using a 0 (none) to 3 (all) scale—five participants reported achieving some of their goals, one reported achieving most of her/his goals, and three reported achieving all of their goals.

During the semi-structured interviews, participants provided more detailed descriptions of aspects of the program they found useful and recommendations for improvement. These are described in Table 2. Overall, the program received largely positive feedback. The program was reported as being “well-structured and engaging,” with benefits ranging from improving diabetic A1C levels, reducing weight, reducing pain, improving mental clarity, and helping to change their perspective on eating. The two most common requests for improving the program in the future included increasing participant contact with other group members with some in-person contact and improving the individualization and quality of the nutritional information, recipes, and examples in the participant workbook.

**Table 2:** Semi-structured interview responses

Theme	Benefits and useful aspects of the program—participant quotes	Least useful aspects of the program and recommendations for change—participant quotes
**Program structure and content**	“well-structured and engaging” “Good program, and I really benefitted from it” “It was a wonderful experience.” “fabulous experience – I learned a lot”	“food logs annoying, but helpful” “would like more biological education about the diet” Would like “more opportunity for discussion outside of group meetings”
**Use of virtual platform**	“better than expected” “Virtual was easier because of transportation” “I liked not having to travel”	“some in-person contact would have made it easier for group members to get to know each other” “less personal feeling” “might be nice to see everyone in-person at least once” “wish we could have met in-person”
**Motivational and behavioral components**	“helped change my perspective on eating” “helpful for achieving my goals” “gained motivation around diet” “goal setting was very helpful … never felt motivated in the past”	“spend more time with each handout and simplify handouts when possible” “some CBT was challenging”
**Group format**	“liked group format for support” “I enjoyed listening to what they had to say”	“some group interactions were frustrating” “Zoom made group more awkward”
**Ketogenic nutrition adherence**	“surprisingly easier than expect” “I enjoyed learning about new type of diet and benefits of lowering carbs” “found new foods I like” “didn’t feel like a diet, just eating differently” “I’m going to live this way the rest of my life”	“didn’t use the recipes” “would like more recipes and nutrition examples” “would like easier to use recipes and macronutrient recommendations that are individualized” “diet too strict” “more guidance on staple foods”
**Benefits/side-effects**	“experienced positive reduction in A1C, from diabetic to pre-diabetic range and less arthritis pain” “mood, weight, mental clarity” “really helped reduce my pain” “mental and physical health” “I always struggled with weight … [I] achieved weight loss goals” “helped my memory” “felt stronger … better energy … less irritable”	“had some constipation” “lost weight unintentionally”

## Discussion

With a growing aging population and increasing rates of Alzheimer’s disease [34], early lifestyle interventions hold significant promise for delaying and slowing the onset of neurodegenerative diseases [1]. Our team used the NIH ORBIT model to adapt existing MI-CBT strategies and previously studied ketogenic nutrition guidelines to test the feasibility and establish proof of concept for a KNA program for older adults with possible MCI. Although our recruitment protocol required modifications, the KNA program demonstrated good feasibility and acceptability in the target population, as well as a clinical signal of effect on cognition. The results of this ORBIT Phase II trial are highly promising and warrant further translation. We discuss the implications of our findings for future pilot studies.

### Feasibility of recruitment protocol

Although our recruitment protocol was successful in identifying participants with possible MCI for this 10-person trial, barriers encountered in our screening procedures suggest that modified approaches may be needed to recruit for larger clinical trials of this kind. First, understanding whether individuals with APOE €4 have distinct responses to nutrition interventions will be important for developing full efficacy data on the KNA program. In our screening sample, however, there was relatively little overlap between individuals who were APOE €4+ and those who had possible MCI. Thus, it may be important to expand inclusion criteria to include individuals who are “at-risk” of developing cognitive impairment, but do not yet have evident cognitive impairment. For example, researchers have begun to identify a pre-MCI stage, which includes both genetic and other risk factors for identifying individuals who are at increased risk for developing MCI and dementia [35]. Alternative approaches may include using a genetic risk score combining multiple risk genes for Alzheimer’s disease and related dementias versus a single allele. These options would allow for the continued use of a precision medicine approach, while expanding inclusion criteria.

A second challenge to recruitment included identifying individuals with probable MCI without other exclusionary health conditions. By expanding our criteria to include any individuals with limited evidence of a change in their memory functioning from their estimated baseline, we were able to identify more than twice the number of participants needed for the current trial. Additionally, this approach to expanding criteria may be more feasible for future dissemination and community uptake. Prior research suggests that ketogenic nutrition may be most effective in the earlier stages of dementia [8, 36]. Thus, future trials may consider alternative screening methods for identifying individuals who are at greatest risk for developing cognitive impairments versus recruiting individuals with existing impairments. For example, use of a dementia risk profile such as the CAIDE risk score [37] combined with brief cognitive assessments and subjective reporting may allow this type of program to be administered as a preventive with broader community reach and application.

### Intervention adherence and retention

Using only once weekly group instruction and the at-home participant workbook, eight of the nine participants were able to detect greater than trace amounts of ketone levels during the intervention period. All participants reported adhering to ketogenic nutrition at least half of the time or greater, with most participants self-reporting very consistent daily adherence. Further, all participants who began the program completed the intervention, with only two participants missing a group meeting. These findings are highly promising in showing that this relatively low-cost group program can produce significant changes in nutrition and engage participants to return weekly. Future trials should examine additional markers of adherence, as some participants struggled to obtain measurable ketone levels despite reporting consistent dietary adherence. It may be especially important to account for metabolic and age differences that may influence how quickly individuals shift into a state of ketosis [38, 39]. Further, additional methods for testing ketone levels may be warranted, as recent studies suggest that urine ketone testing may not be sensitive to mild ketosis [40]. Finally, to enhance retention and engagement, future trials should examine differences between in-person and virtual meetings.

### Clinical signal of effect on cognition

Encouragingly, even with a sample of only nine participants, a statistically and clinically significant improvement was observed from baseline to week 6 of the program on the cognitive assessment. These clinically significant improvements offer additional support for the intervention’s readiness to move into the next phase of the NIH ORBIT model for intervention development. Importantly, these future studies must evaluate whether the MI and CBT strategies were additive to adherence and subsequent outcomes associated with the KNA program. Future studies may also benefit from the addition of long-term assessment time points to evaluate whether adherence to ketogenic nutrition and gains in cognitive performance persist long-term.

### Qualitative acceptability of the program

Qualitative data collected during participant interviews provided valuable information about aspects of the program that participants enjoyed and areas for improvement in future trials. Flexibility in design and development at this stage are vital in the NIH ORBIT model, as this information can be used for further intervention refinement. In general, participants reported enjoying the overall program, finding goal setting and motivational components useful, and experiencing a range of mental and physical benefits from the program. Participants consistently reported a desire for some in-person components of the program, although the virtual platform was described as acceptable and easily accessible. Participants also made recommendations for improving the quality of the ketogenic nutrition information and participant workbook to enhance individualization and a greater number of recipes and examples.

### Limitations

Given the early phase of this single-arm trial, there are some inherent limitations that must be considered when interpreting the findings. First, although relatively diverse, our sample size was small and there was no comparison group. Thus, closer analysis of individuals who may benefit most from this type of nutrition was not possible. Further, without a comparison group, we were not able to assess which components of the treatment may be most beneficial for improving adherence to ketogenic nutrition. Future pilot trials should assess how the MI and CBT components of the program may contribute to adherence beyond the group setting and structured nutritional guidance. Finally, it is important to note that this trial was retrospectively registered with clinicaltrials.gov.

## Conclusions and future directions

The current study demonstrated feasibility of the intervention protocol through high retention and adherence and established the proof of concept for the KNA program based on the NIH ORBIT model’s recommended milestones. An important next step in scaling up this program will involve the inclusion of key stakeholders to identify appropriate community outlets for reaching high-risk older adults. Further, we will continue to develop the program materials to allow training and administration of the program by community members in order to increase its potential reach. Overall, the program is ready to move to the next phase of intervention development—pilot testing (IIb & c), where our team will work with community partners to evaluate the efficacy of our protocol in a community setting.

## Data Availability

The datasets generated and analyzed during the current study are not publicly available due to the small sample size and potential for loss of confidentiality of participant information through data sharing. Fully de-identified data or portions of the dataset may be available from the corresponding author on reasonable request.
